# HER2 Alterations in Squamous Cell Lung Cancer: Biology, Therapeutic Landscape, and Emerging Precision Approaches

**DOI:** 10.3390/cancers18132121

**Published:** 2026-06-30

**Authors:** Dina Elantably, Isabella Meerzaman, Alicia Y. Hou, Ahmed Abdelhakeem, Yanyan Lou

**Affiliations:** 1 Division of Haematology and Oncology, Department of Internal Medicine, Mayo Clinic, Jacksonville, FL 32224, USA; hou.alicia@mayo.edu (A.Y.H.); abdelhakeem.ahmed@mayo.edu (A.A.); lou.yanyan@mayo.edu (Y.L.); 2 DeBusk College of Osteopathic Medicine, Lincoln Memorial University, Harrogate, TN 37752, USA; isabella.meerzaman@lmunet.edu

**Keywords:** HER2, squamous cell lung cancer, NSCLC, ERBB2, antibody/drug conjugates, precision oncology, HER2 biomarkers, targeted therapy, next-generation sequencing (NGS), immunohistochemistry (IHC)

## Abstract

Squamous cell lung cancer (SqCLC) accounts for 20–30% of non-small cell lung cancer (NSCLC) and remains associated with a poorer prognosis compared with adenocarcinoma. Despite advances in treatment, 5-year overall survival for advanced (stage IV) disease remains below 10–15%. Unlike non-squamous NSCLC, SqCLC is characterized by a high tumor mutational burden and complex genomic landscape dominated by alterations in tumor suppressor genes and lineage survival pathways including TP53, CDKN2A, PIK3CA, FGFR1, SOX2, and the NFE2L2/KEAP1 oxidative stress pathway, as well as dysregulation of the NOTCH signaling pathway, but it harbors relatively few actionable oncogenic drivers, resulting in limited treatments for targeted therapy. HER2 alterations can occur by multiple mechanisms, including activating mutations, gene amplifications, and protein overexpression. They comprise a very small percentage of NSCLC, with HER2 mutations reported in approximately 1–3% and HER2 amplifications observed roughly in 2–4%. While HER2 alterations are well characterized in lung adenocarcinoma, the prevalence, genomic context, and clinical significance of HER2 alterations in SqCLC remain incompletely defined. Advances in next-generation sequencing have led to improved ability to detect HER2 alterations and facilitated the development of HER2 targeted therapies. Available treatments for advanced/metastatic SqCLC have been historically limited to platinum-doublet chemotherapy, with immune checkpoint inhibitors such as anti-PD-1/PD-L1 newly emerging in the past decade. Selective HER2 tyrosine kinase inhibitors and HER2 antibody/drug conjugates have shown improved efficacy in HER2-altered NSCLC as shown in DESTINY-LUNG02 and BEAMION LUNG-1 trials; however, most of the enrolled patients had non-squamous histology, with minimal or no SqCLC-specific efficacy data reported. Future progress in HER2-altered SqCLC will require inclusion of SqCLC in HER2 basket trials, incorporation of comprehensive molecular profiling and standardized HER2 testing in squamous histology. This review summarizes the current state of knowledge of HER2 biology in SqCLC and highlights areas for future directions for precision oncology in SqCLC.

## Simple Summary

HER2 dysregulation is well recognized in lung adenocarcinoma, but its significance in squamous cell lung cancer remains underexplored. Recent genomic datasets reveal that HER2 mutations, amplifications, and protein overexpression do occur in squamous non-small cell lung cancer (NSCLC), suggesting potential therapeutic relevance. With the rapid development of HER2-directed therapies, including antibody drug conjugates and next-generation tyrosine kinase inhibitors, it is increasingly important to clarify their roles in this histologic subtype. This review will summarize the biology and prevalence of HER2 alterations in squamous NSCLC, discuss diagnostic considerations, evaluate existing clinical data, and highlight future precision medicine strategies for this rare molecular subset.

## 1. Introduction

Lung cancer remains the leading cause of cancer-related mortality worldwide, with recent GLOBOCAN 2022 estimates reporting approximately 2.48 million new cases and 1.8 million deaths globally [1]. Non-small cell lung cancer (NSCLC) makes up about 83% of all lung cancer diagnoses and includes squamous cell carcinoma (SqCLC), adenocarcinoma, and large cell carcinoma [2]. Squamous cell lung cancer (SqCLC) represents approximately 20–30% of NSCLC and remains a clinically challenging subtype, with poorer survival outcomes than lung adenocarcinoma despite advances in systemic therapy [3]. Although overall survival (OS) in NSCLC has improved substantially in recent years, largely driven by the implementation of low-dose computed tomography (CT) screening and advances in targeted therapy and immunotherapy, with 5-year OS rising from 13–15% to 26–28% in the early to mid 1990s, advanced SqCLC continues to be associated with limited therapeutic options and a persistently poor prognosis, with median overall survival generally remaining approximately 12–18 months with chemo-immunotherapy [4,5]. The incorporation of molecular profiling has fundamentally transformed the treatment landscape of NSCLC by enabling the identification of actionable oncogenic drivers and the development of targeted therapies. In lung adenocarcinoma specifically, approximately 40–50% of patients in Western populations harbor actionable oncogenic alterations, including EGFR, ALK, ROS1, RET, MET, BRAF, KRAS G12C, NTRK, NRG1, and HER2 (ERBB2), with EGFR mutations occurring at higher frequencies in Asian populations, approaching 40–50% in some series [6,7]. In contrast, available large-scale genomic datasets indicate that actionable genomic alterations remain substantially less common in SqCLC than in non-squamous NSCLC, with estimates ranging from 1 to 10% across different datasets, although precise estimates vary according to the molecular targets included and the extent of genomic profiling performed [8,9,10].

SqCLC is characterized by a high degree of genomic complexity and high tumor mutational burdens, largely attributable to tobacco-related carcinogenesis. Comprehensive genomic analyses from The Cancer Genome Atlas (TCGA) have demonstrated a substantial burden of somatic mutations, along with widespread chromosomal instability, including frequent structural rearrangements, focal amplifications, and copy number alterations [11]. Unlike lung adenocarcinoma, SqCLC is predominantly driven by loss-of-function alterations in tumor suppressor genes rather than targetable oncogenic drivers. Recurrent genomic events include frequent alterations in TP53 (occurring in >80–90% of SqCLC cases), as well as CDKN2A/RB1 pathway alterations, which occur in approximately 72% of SqCLC cases and contribute to cell cycle dysregulation [11]. Additionally, alterations in oxidative stress response pathways, particularly the NFE2L2–KEAP1 axis, are observed in approximately 20–30% of tumors and are associated with therapeutic resistance and poor clinical outcomes [11]. Dysregulation of the PI3K/AKT/mTOR signaling pathway, often through PIK3CA mutations or amplifications, as well as PTEN loss, further contributes to tumor proliferation, survival, and therapeutic resistance in SqCLC [11]. Amplification of chromosome 3q represents a hallmark and early event in squamous tumorigenesis, encompassing key lineage-survival and oncogenic drivers such as SOX2, TP63, and PIK3CA, and is present in the majority of SqCLC cases [11,12]. FGFR1 amplification, identified in approximately 15–20% of SqCLC tumors, has also historically represented a major therapeutic target of interest in squamous histology; however, clinical translation of FGFR-targeted therapy in FGFR1-amplified SqCLC has shown only modest response rates across multiple clinical trials [12,13]. Collectively, this complex and heterogeneous genomic landscape poses significant challenges for the development of effective targeted therapies in SqCLC.

Human epidermal growth factor receptor 2 (HER2/ERBB2) has emerged as a promising therapeutic target in NSCLC and has gained increasing attention in the era of precision oncology. HER2 alterations arise through multiple distinct mechanisms, including mutation, gene amplification, and protein overexpression, each of which may have different biological and therapeutic implications. In lung adenocarcinoma, HER2/ERBB2 mutations are found in approximately 2–4% of unselected lung adenocarcinoma and define a molecularly distinct subset of disease [14,15,16]. HER2 amplification in lung adenocarcinoma is reported in approximately 1–3% of cases, although prevalence estimates vary depending on the detection method, including fluorescence in situ hybridization (FISH), immunohistochemistry (IHC), and NGS-based copy number assessment [17,18]. HER2-directed therapies have recently demonstrated improved response rates and durable clinical benefit in pretreated patients. However, the prevalence and biological significance of HER2 alterations in SqCLC remain incompletely characterized, with available studies suggesting that these alterations are rare, including HER2 mutations reported in approximately 0.4–1% of cases and HER2 amplification in roughly 2–4% of tumors. Their true prevalence may also be underestimated due to the historically limited use of comprehensive genomic profiling in squamous histology [19,20,21]. Furthermore, the genomic context of HER2 alterations in SqCLC differs substantially from that in adenocarcinoma, potentially influencing therapeutic sensitivity and resistance mechanisms. HER2 tyrosine kinase domain (TKD) mutations in lung adenocarcinoma are strongly enriched in women who have never smoked, and may occur more frequently in Asian populations, mirroring the epidemiologic profile observed in EGFR-mutant lung cancer. In contrast, SqCLC is predominantly associated with smoking, rarely harbors TKD mutations and instead appears relatively enriched for extracellular domain (ECD) mutations, which are associated with tobacco-related mutations [22]. The vast majority of clinical trials evaluating HER2-directed therapies are focused primarily on non-squamous populations, therefore creating a gap in evidence for squamous histology [23].

This narrative review summarizes the current understanding of HER2 alterations in SqCLC, including HER2 biology, genomic landscape, diagnostic modalities, and emerging HER2-targeted therapeutic strategies, while also proposing a precision oncology framework for HER2-altered SqCLC. A literature review was conducted using PubMed, Embase, major oncology conference proceedings, and relevant clinical trial databases through March 2026 using search terms including ‘HER2’, ‘ERBB2’, ‘squamous NSCLC’, ‘HER2 amplification’, ‘HER2 mutation’, and ‘antibody-drug conjugates’.

## 2. Biology of HER2 in Lung Cancer

### 2.1. HER2 Structure and Signaling

HER2 is a human epidermal growth factor receptor 2, encoded by the ERBB2 gene, with a molecular size of roughly 185 kDa. It is a transmembrane receptor present at the cell surface as a monomer, consisting of an extracellular domain (ECD) composed of four subdomains (I–IV), a transmembrane domain, and an intracellular tyrosine kinase domain (TKD) [24]. The extracellular domain contains the receptor dimerization interface within domain II, where recurrent activating mutations such as S310F and G309E have been identified. Unlike other HER/ERBB family members, HER2 functions as an orphan receptor without a known ligand and adopts a fixed dimerization-competent extracellular conformation. This structural configuration makes HER2 the preferred heterodimerization partner for other HER family receptors, including EGFR (HER1), HER3, and HER4 [25]. This heterodimerization phosphorylates HER2, while HER2 enhances transphosphorylation of other HER family members; thus, creating docking sites for downstream signaling molecules [24]. Among the HER family, HER2–HER3 is considered the most potent oncogenic dimer, as HER3 possesses impaired intrinsic kinase activity (pseudokinase activity) but strongly couples to PI3K/AKT pathway activation [26]. The major downstream signaling pathways activated by HER2 include the PI3K/AKT, RAS/RAF/MEK/MAPK, and JAK/STAT signaling pathways, which collectively regulate cell proliferation, survival, differentiation, and therapeutic resistance. Additional signaling through phospholipase C-gamma (PLC-γ) and NF-κB pathways may also occur in a context-dependent manner [27]. HER2 receptor trafficking and internalization have important therapeutic implications. Antibody drug conjugates (ADCs) rely on receptor-mediated internalization and lysosomal payload release following HER2 binding [28]. Unlike many receptor tyrosine kinases, HER2 is relatively endocytosis resistant and remains stabilized at the cell surface through mechanisms involving HSP90-mediated receptor recycling. Following internalization, ADCs traffic through endosomal and lysosomal compartments, where linker cleavage and intracellular payload release occur [28,29].

### 2.2. Mechanisms of HER2 Dysregulation

#### 2.2.1. HER2 Mutation

Approximately 70–80% of HER2 mutations occur within the tyrosine kinase domain (TKD), although mutations have also been described in the extracellular domain and transmembrane domain [16]. The most common type of mutation is the in-frame insertion in exon 20, particularly the Y772_A775dupYVMA variant. This mutation enhances HER2 kinase activity through enhanced autophosphorylation of the receptor and transphosphorylation of other HER family members, including EGFR and HER3, thus facilitating downstream activation of the MAPK and PI3K-AKT signaling pathways [30].

Other Exon 20 mutations that can promote constitutive activation of TKD and reduce sensitivity to HER2 inhibitors such as lapatinib include G776delinsVC, P780_Y781insGSP, L775S, V777L, and D769H/Y [31].

Mutations that activate HER2 through extracellular domain include S310F and G309E [30]. In contrast, Ile655Val represents a germline polymorphism rather than a somatic driver mutation and has been associated with enhanced HER2 dimerization and receptor activation through structural alterations within the transmembrane domain [32]. Representative HER2 exon 20 and extracellular domain mutations, associated biologic effects, and therapeutic implications are summarized in Table 1.

#### 2.2.2. HER2 Amplification and Overexpression

HER2 gene amplification involves an increased copy number of the ERBB2 gene located on chromosome 17q12, often resulting in high-level amplification with more than 10 gene copies per nucleus. Such amplification drives receptor overexpression and constitutive, ligand-independent downstream signaling through the PI3K/AKT and MAPK pathways [42,43].

The molecular mechanisms of HER2 amplification can be various. These include chromoanasynthesis, amplification driven by inverted duplications and breakage–fusion–bridge cycles, and extrachromosomal DNA (ecDNA) amplification. ecDNA is believed to increase the intratumoral heterogeneity of HER2 via random segregation of HER2 during cell division, and thus potentially contributes to the variable HER2 expression [44,45,46].

**Figure 1:**
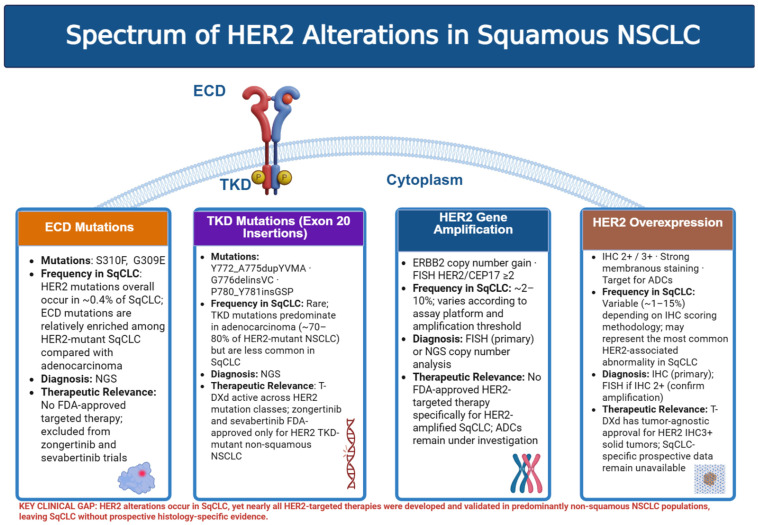
Spectrum of HER2 alterations in squamous cell lung cancer (SqCLC). HER2 dysregulation occurs through multiple mechanisms including extracellular domain mutations, tyrosine kinase domain mutations (most commonly exon 20 insertions), gene amplification, and protein overexpression. These alterations differ in frequency, biological significance, and therapeutic implications. ECD HER2 mutations are relatively enriched in SqCLC compared with adenocarcinoma and are associated with smoking-associated genomic features, higher tumor mutational burden, and increased co-mutation frequency. Created in BioRender. Elantably, D. (2026) https://BioRender.com/vc7n88f. ECD = extracellular domain; TKD = tyrosine kinase domain; SqCLC = squamous cell lung carcinoma; NGS = next-generation sequencing; IHC = immunohistochemistry; FISH = fluorescence in situ hybridization; ADC = antibody-drug conjugate; T-DXd = trastuzumab deruxtecan.

HER2 protein overexpression, frequently defined as IHC 2+ or 3+, can occur separately and is not necessarily indicative of true gene amplification [47]. However, unlike breast and gastroesophageal cancers, there is currently no standardized or validated immunohistochemistry scoring system for NSCLC, which is an important diagnostic limitation. Mechanisms of HER2 overexpression include chromosome 17 polysomy, transcriptional upregulation due to epigenetic modulation, structural variations that target enhancer elements, and post-translational regulation, including recycling of the HER2 receptor and accumulation of the HER2 protein on the membrane due to PKCα signaling activity [48]. Figure 1 outlines the spectrum of HER2 alterations in SqCLC.

## 3. Prevalence and Genomic Landscape of HER2 Alterations in Squamous NSCLC

### 3.1. HER2 Mutations in Squamous NSCLC

Large sequencing cohorts demonstrate that HER2 alterations are substantially less frequent in SqCLC compared with non-squamous NSCLC. In one of the largest available datasets from Foundation Medicine comprising 93,465 NSCLC samples, activating HER2 (ERBB2) alterations were identified in 2.3% of squamous tumors compared with 4.3% of non-squamous tumors, largely driven by differences in HER2 mutation frequencies (0.4% vs. 2.0%, respectively) [20]. Table 2 summarizes the prevalence of HER2 alterations in SqCLC by study, assay, and alteration type.

The mutational profile in squamous NSCLC differs from that observed in adenocarcinoma. In HER2-mutant NSCLC overall, tyrosine kinase domain (TKD) mutations predominate (70–80% of all HER2 mutations), these are strongly associated with adenocarcinoma histology, female sex, and never smoking status. In contrast, when HER2 mutations occur in SqCLC, available evidence suggests relative enrichment for extracellular domain (ECD) mutations, particularly S310F/Y alterations, which occur within the receptor dimerization interface and may represent a biologically distinct subtype of HER2-driven disease [49].

Patients with HER2 mutant squamous cell tumors are more likely to have a history of tobacco exposure, and show somatic mutations consistent with mutational signature associated with smoking [50]. These tumors often exist within a complex genomic background with multiple other co-mutations, involving TP53, EGFR, KRAS, STK11, KEAP1, and SMARCA4 [50]. Furthermore, tumors with ECD mutations have increased tumor mutation burden compared to tumors with kinase domain alterations, with median TMB values of 11.1 mutations/Mb vs. 5.2 mutations/Mb, respectively (*p* < 0.003), representing an approximately twofold higher TMB in ECD-mutant NSCLC [16]. Although HER2 mutations are generally considered mutually exclusive with some oncogenic driver mutations, this mutual exclusivity applies primarily to TKD mutations. ECD mutations show a strikingly different pattern with high rates of co-mutations [49]. Similarly, co-occurring genomic alterations are more common in squamous NSCLC [20].

Due to the rarity of HER2 mutant SqCLC, prognostic data are limited and largely hypothesis generating. Because available data regarding HER2 alterations in SqCLC are derived from heterogeneous cohorts employing variable assay methodologies and reporting practices, we summarized major genomic studies according to cohort characteristics, alteration definitions, prevalence estimates, clinicopathologic associations, and reported outcomes (Table 2). Emerging evidence suggests that mutation domain may influence clinical outcomes. In the initial AACR Project GENIE analysis, patients harboring extracellular domain (ECD) mutations demonstrated shorter progression-free survival (PFS) with first-line chemotherapy compared with tumors harboring tyrosine kinase domain (TKD) mutations (HR 2.4, *p* = 0.04) [50]. However, subsequent analyses from the Flatiron Health cohort by Stockhammer et al. (2026) showed improved outcomes among patients receiving first-line chemo-immunotherapy, with ECD-mutant tumors exhibiting longer 12-month PFS compared with TKD mutant tumors (35% vs. 18%; HR 0.72, *p* = 0.026), a finding that persisted in multivariate analyses and may reflect the higher tumor mutational burden and smoking-associated phenotype characteristic of ECD mutant tumors [16]. Co-occurring genomic mutations may further influence prognosis. STK11 and KEAP1 co-mutations were associated with inferior outcomes, with shorter PFS observed among tumors harboring STK11 (HR 3.5, *p* = 0.03) or KEAP1 alterations (HR 3.9, *p* = 0.002) [50]. Although broader NSCLC datasets suggest that HER2 mutant tumors have intermediate survival outcomes compared with other oncogenic subsets, SqCLC-specific overall survival data remain limited due to small patient numbers The only prospective data specifically addressing HER2 mutations in SqCLC come from the LUX-Lung 8 secondary analysis. Patients with HER2 mutant tumors treated with afatinib had dramatically better outcomes compared to Erlotinib (HR 0.06 for both PFS and OS). However, subsequent in vitro validation showed that the majority of these HER2 mutations were non-oncogenic, raising questions about whether these mutations were truly driving the clinical benefit [49].

HER2 ECD mutations in SqCLC represent an important therapeutic gap as currently FDA-approved HER2-selective tyrosine kinase inhibitors (TKIs), including zogertinib and sevabertinib, were developed primarily for TKD-mutant non-squamous NSCLC and have showed limited and inconsistent activity in non-TKD mutations [33,34]. Mechanistically, this may reflect the fact that ECD mutations alter receptor dimerization and extracellular structure rather than the intracellular catalytic domain targeted by these agents. In contrast, antibody drug conjugates (ADCs) are independent of kinase domain inhibition. Trastuzumab deruxtecan has demonstrated activity across multiple HER2 mutation classes and currently carries a broad approval for HER2-mutant NSCLC without restriction by mutation domain or histology [19]. Additional evidence from studies evaluating ado-trastuzumab emtansine and Trastuzumab rezetecan suggests that therapeutic activity may extend across kinase domain, transmembrane, and extracellular domain alterations [51,52].

**Table 2:** Prevalence of HER2 alterations in squamous NSCLC by study, assay, and alteration type.

Study	N (Total/SCC)	Assay Platform	Alteration Definition	Prevalence (SCC)	Smoking Status	Key Co-Mutations	Clinical Outcome
Foundation Medicine [20]	85,704 tissue (SCC subset included)	CGP (FoundationOne)	ERBB2 mut (activating SNVs/indels)	0.4% in SCC vs. 2.0% in non-SCC	NR	Mutually exclusive with KRAS, EGFR, ALK, BRAF, ROS1, RET, MET	NR
Foundation Medicine [20]	85,704 tissue	CGP (FoundationOne)	ERBB2 amp (≥ploidy + 3)	Part of 2.3% total ERBB2 alt in SCC (amp + mut combined)	NR	NR	NR
Caris Life Sciences [53]	52,690 NSCLC	NGS (592-gene/WES) + IHC	HER2 mut; HER2 amp (CN > 6); HER2 IHC 2+	670 mut, 400 amp, 272 IHC 2+ (histology-specific breakdown NR)	Female predominance for mut (59.7%), smoking NR	HER2 amp: TP53 90%, EGFR 10%, SMARCA4 12%, TMB-H 47%; HER2 IHC 2+: KRAS 33%, KEAP1 21%	HER2 mut OS 22.0 mo; HER2 amp shorter OS vs. mut
Hirsch et al. (2002) [54]	238 NSCLC (SCC subset)	IHC (HercepTest) + FISH	IHC 2+/3+	1% in SCC vs. 35% in adeno	NR	NR	No survival difference (IHC 2+/3+ vs. 0/1+)
Grob et al. (2012) [55]	590 NSCLC (adeno, SCC, large cell)	IHC + FISH	ERBB2 amp (FISH, gene clusters)	3% overall; propensity to high-grade adeno (12%); SCC-specific rate low	NR	NR	NR; intratumoral heterogeneity in 4/10 highly amplified
Pelosi et al. (2005) [56]	345 Stage I NSCLC (all histologies)	IHC + FISH	IHC any positivity; amp (HER2/CEP17 ≥ 2.0)	IHC+ 23% overall; amp in 2 SCC (1 high-level, 1 low-level)	NR	NR	No prognostic impact
Odintsov et al. (2024) [57]	5769 NSCLC (5075 pts)	NGS (OncoPanel)	High-level ERBB2 amp (≥6 copies)	0.9% of adeno; SCC-specific rate NR but lower	Smokers: sole driver; never smokers: co-alteration	Two genomic subsets: sole driver (smokers) vs. co-alteration (never smokers)	T-DXd active in preclinical models and case reports
Stockhammer et al. (2026) (GENIE + FH) [16]	483 + 286 ERBB2-mut NSCLC	CGP (GENIE/FH)	ERBB2 mut by domain (TKD, ECD, TMD/JMD)	SCC: 7% of ECD-mut vs. 1% of TKD-mut tumors	ECD-mut: 100% smokers (n = 39 subset)	ECD: EGFR 32%, KRAS 17%, STK11 23%, KEAP1 23%; TMD/JMD: PIK3CA 24%	ECD-mut: longer PFS with chemo-immuno vs. TKD (HR 0.72)
Ninomiya et al. (2019) (HER2-CS) [58]	1126 NSCLC (all histologies)	IHC + FISH + Sanger sequencing	IHC 3+; IHC 2+/FISH+; exon 20 ins mut	IHC 3+ 3.0%, IHC 2+/FISH+ 3.0%, mut 2.9% (overall; SCC-specific NR)	IHC 3+ and IHC 2+/FISH+: male, smokers; mut: female, never smokers	IHC 3+ and mut entirely exclusive	Worse prognosis vs. EGFR+/ALK+
Li et al. (2016) [59]	175 adeno only	FISH (HER2/CEP17 ≥ 2.0) + fragment analysis/Sanger + IHC	Amp, mut, IHC 2+/3+	Amp 3%, mut 3%; no overlap; IHC 2+/3+ 0% (n = 25 tested)	NR	Mut and amp mutually exclusive	NR
Suzuki et al. (2015) [60]	1275 NSCLC (146 SCC)	HRM + CISH + IHC	HER2 mut (exon 20 ins); amp (CISH)	Mut: 0% in SCC (all 46 mut in adeno); amp in ~50% of mut cases	Mut: never smokers	Mut exclusive of other drivers	Mut: unfavorable OS in invasive adeno

Abbreviations: SCC, squamous cell carcinoma; NSCLC, non-small cell lung cancer; CGP, comprehensive genomic profiling; NGS, next-generation sequencing; WES, whole-exome sequencing; IHC, immunohistochemistry; FISH, fluorescence in situ hybridization; CISH, chromogenic in situ hybridization; CN, copy number; amp, amplification; mut, mutation; SNV, single nucleotide variant; indel, insertion/deletion;; OS, overall survival; PFS, progression-free survival; TMB-H, tumor mutational burden—high; TKD, tyrosine kinase domain; ECD, extracellular domain; TMD/JMD, transmembrane/juxtamembrane domain; NR, not reported. Histology-specific prevalence values are reported when available; otherwise, data reflect overall NSCLC populations. Because HER2 alterations in SqCLC are rare, several studies did not report dedicated squamous subgroup analyses or outcomes separately.

### 3.2. HER2 Amplification and Overexpression in Squamous NSCLC

HER2 amplification are also uncommon in SqCLC, although frequencies reported may vary depending on the specific assay used and/or copy number thresholds. Across several large series, HER2-amplified tumors occurred in approximately 2–10% of cases [54,61]. The broad range of reported HER2 amplification prevalence likely reflects substantial methodological heterogeneity across studies, including differences in patient selection (resected vs. advanced-stage cohorts), assay methodology (FISH, CISH, DISH, or NGS-based copy number assessment), and amplification definitions, with some studies applying conventional HER2/CEP17 ratio thresholds ≥ 2.0 while others use more stringent criteria such as HER2/CEP17 ratios ≥ 5 or absolute copy number thresholds ≥ 10. These methodological differences likely account for much of the observed variability in reported SqCLC prevalence estimates (Table 2). In a comprehensive analysis of 590 lung cancers, HER2 amplification was seen in 3% of tumors overall with significantly more HER2 amplifications identified among high-grade adenocarcinomas (12%) compared to SqCLC [61]. Similarly, in an analysis of 157 resected SqCLC, HER2 amplification was detected in 9.6% of tumors; however, very few tumors demonstrated high-level amplification, defined in this study as ≥6 HER2 copies and/or HER2/CEP17 ratio ≥ 5 [54,55].

HER2 protein overexpression is also rare in SqCLC. In one study of 238 NSCLC cases, moderate-to-high membranous staining (IHC 2+/3+) was found in only 1% of squamous tumors, compared with 35% of adenocarcinomas [57]. Marked (3+) overexpression seen in NSCLC is rare among histologies but is particularly rare in SqCLC [57]. Interpretation of HER2 immunohistochemistry in SqCLC may be particularly challenging, as squamous tumors frequently demonstrate heterogeneous staining patterns, including cytoplasmic and basal localization, which may be misinterpreted or inconsistently scored. This further emphasizes the lack of validated HER2 scoring criteria in SqCLC and supports the need for histology-specific standardization.

Unlike the strong correlation between HER2 amplification and clinical significance seen in breast cancer, the association between HER2 gain and protein overexpression in lung cancer is less consistent and largely determined by the level of HER2 amplification [54]. Although high-level HER2 amplification correlates strongly with protein overexpression, there was little correlation seen between low-level HER2 gains and IHC results [54]. In a study of 51 NSCLCs, only 4% demonstrated both marked (3+) protein expression and gene amplification, while a large percentage of tumors exhibited HER2 gain with no corresponding protein expression [57]. The increased copy number seen appears to be due to chromosome 17 aneusomy, rather than true focal amplification [61].

For HER2-amplified SqCLC, the genomic background frequently includes TP53 mutations, CDKN2A deletions, and SOX2 amplification [62]. CDKN2A deletions co-occur with both HER2 amplification and mutations, whereas BRAF mutations are mutually exclusive of HER2 changes [53].

The prognostic significance of HER2 overexpression and amplification in SqCLC remains uncertain. Analysis of pooled data from a meta-analysis of 40 studies encompassing 6135 NSCLC patients showed no significant association of HER2 overexpression with overall survival in the SqCLC subgroup (HR 0.87; 95% CI 0.61–1.25), although the number of SqCLC patients contributing to this estimate was not separately reported and was likely small given the wide confidence interval which underscores the limited statistical power of this subgroup analysis [63]. High-level HER2 amplification has been associated with inferior disease-free survival in lung adenocarcinoma but not specifically SqCLC. TP53 co-mutation in patients with HER2-altered NSCLC has been associated with inferior outcomes in several studies, although these findings are not SqCLC-specific [34,64,65].

Notably, HER2-low expression (IHC 1+ or 2+ without gene amplification) is an emerging category of clinical interest across solid tumors. HER2-low expression has been identified in approximately 47% of lung cancers, and quantitative immunofluorescence studies suggest that over 63% of NSCLC cases harbor detectable HER2 protein expression, with many in a range comparable to breast cancer IHC1+/2+ [66,67]. While the HER2-low paradigm has transformed treatment selection in breast cancer through the demonstrated efficacy of trastuzumab deruxtecan (T-DXd) in HER2-low disease, its relevance to NSCLC and SqCLC in particular remains investigational. Whether HER2-low expression carries independent prognostic or predictive significance in SqCLC is an important unanswered question that warrants prospective studies.

## 4. Diagnostic Considerations for HER2 in Squamous NSCLC

### 4.1. Detection of HER2 Mutations

**Figure 2:**
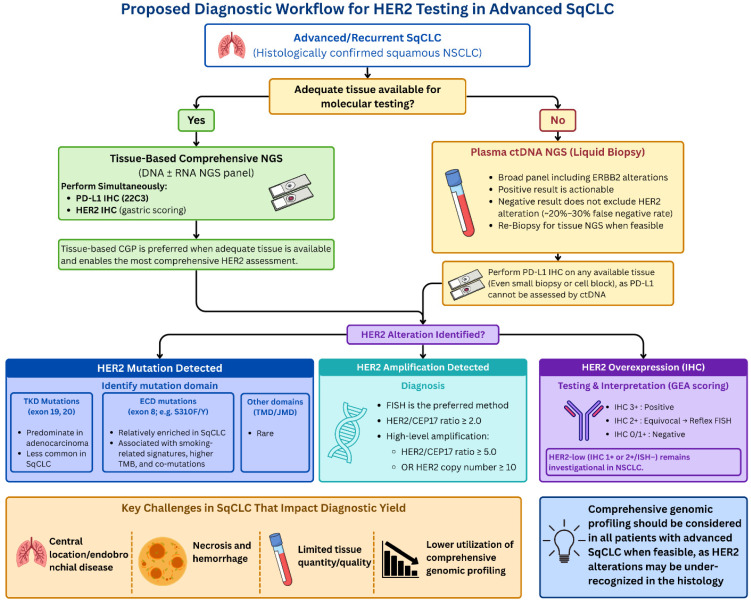
Proposed diagnostic workflow for identification of HER2 alterations in advanced squamous non-small cell lung cancer (SqCLC). Abbreviations: CGP, comprehensive genomic profiling; ctDNA, circulating tumor DNA; ECD, extracellular domain; ERBB2, erb-b2 receptor tyrosine kinase 2; FISH, fluorescence in situ hybridization; GEA, gastroesophageal adenocarcinoma; HER2, human epidermal growth factor receptor 2; IHC, immunohistochemistry; ISH, in situ hybridization; JMD, juxtamembrane domain; NGS, next-generation sequencing; NSCLC, non-small cell lung cancer; PD-L1, programmed death-ligand 1; SqCLC, squamous cell lung cancer; TKD, tyrosine kinase domain; TMB, tumor mutational burden; TMD, transmembrane domain.

The use of next-generation sequencing (NGS) for detecting HER2 mutations in NSCLC has been the standard, as it can identify multiple types of point mutations, insertions, duplications, and gene amplification in one assay. Current NCCN guidelines endorse testing with an NGS multigene panel in patients with advanced NSCLC [68]. Figure 2 summarizes a proposed diagnostic workflow for identification of HER2 alterations in advanced squamous non-small cell lung cancer (SqCLC).

DNA-based NGS detects HER2 mutations and provides a cumulative method to characterize the molecular heterogeneity of HER2 variants [68]. RNA sequencing could also be utilized to aid in determining structural gene variations such as fusions and splicing variants that may be missed by DNA sequencing alone. Therefore, the NCCN recommends considering RNA-based sequencing when DNA results fail to identify any actionable driver alterations [68]. Recently, a combination of RNA and DNA sequencing increased detection of actionable mutations by approximately 15% compared with DNA sequencing alone [69].

Tissue NGS has shown a high level of analytical accuracy for HER2 mutation detection with a positive percent agreement to clinical trial reference assays between 98 and 100% and with negative percent agreement of 100% [70]. Liquid biopsy is another method to determine HER2 mutation status, particularly if tissue is unavailable. There were no statistical differences in mutation frequencies between plasma- and tissue-based sequencing for detection of HER2 mutations, with reported frequencies of 2.2% in ctDNA samples and 2.1% in tissue samples [71]. Thus, the NCCN supports the use of either tissue or plasma testing, or both concurrently, to provide a fast turnaround time and to maximize the identification of actionable alterations [68].

SqCLC presents several unique diagnostic challenges that may contribute to underdetection of HER2 alterations. Compared with adenocarcinoma, squamous tumors more frequently arise centrally, involve endobronchial structures, and exhibit extensive necrosis, hemorrhage, and crush artifact, all of which can compromise tissue quality and reduce the amount of material available for molecular testing. Furthermore, diagnostic procedures are often performed primarily to establish histology rather than obtain tissue for comprehensive biomarker assessment, increasing the likelihood of exhausted specimens. These factors may contribute to lower rates of comprehensive genomic profiling in SqCLC and may partially explain the historically lower reported prevalence of actionable genomic alterations in this population. When tissue quantity or quality is limited, complementary liquid biopsy approaches may help improve detection of clinically relevant HER2 alterations. The use of any minimally invasive biopsy procedures (e.g., bronchoscopy biopsies; EBUS transbronchial needle aspirations (TBNA)) may result in insufficient yield for complete genomic profiling and intratumoral heterogeneity may not be adequately sampled by small biopsy specimens [55,72,73]. Negative results should be interpreted cautiously, given that low levels of tumor fraction in the tissue or low levels of ctDNA in plasma can yield false negative results.

### 4.2. Detection of HER2 Amplification and Overexpression

A major limitation in HER2 testing for NSCLC is the absence of an FDA-approved HER2 immunohistochemistry companion diagnostic, resulting in reliance on scoring extrapolated from breast and gastroesophageal adenocarcinoma (GEA). Currently, HER2 expression assessment in NSCLC most commonly utilizes GEA-derived scoring criteria rather than breast cancer criteria; however, these methodologies have not been specifically validated in SqCLC. According to GEA guidelines, HER2 IHC3+ expression is defined by strong complete basolateral or lateral membrane staining involving ≥ 10% of the total tumoral area stained [74]. This differs from breast cancer criteria, where circumferentially intense membrane staining is required to classify tumors as IHC3+. The GEA system has been used for NSCLC because lung malignancies frequently show incomplete or heterogeneous membranous staining patterns that are more similar to gastric cancer than they are to breast cancer [75]. However, interpretation of HER2 immunohistochemistry in SqCLC presents additional challenges, as squamous tumors frequently exhibit heterogeneous, cytoplasmic, and basal staining patterns that may complicate interpretation and contribute to inter-observer variability. Re-scoring NSCLC samples using GEA criteria has been shown to increase HER2 positivity rates and enhance concordance with HER2 gene amplification testing (from 87.5% to 93.8%) [75]. Nevertheless, the limitation of inter-observer variability persists, and no specific antibody clone is universally recommended for assessment of HER2 in lung cancer [76]. Emerging HER2 expression categories, including HER2-low (IHC 1+ or IHC 2+/ISH-negative) and HER2-ultralow expression states (minimal or focal membrane staining insufficient for conventional positivity), have recently gained interest across multiple tumor types because antibody-drug conjugates may retain activity despite lower HER2 expression levels through bystander effects [47]. However, unlike breast cancer, the clinical significance of HER2-low and HER2-ultralow expression in NSCLC remains poorly defined, and there are currently insufficient data to determine whether lower-expression SqCLC tumors derive meaningful benefit from HER2-directed therapies [47,66]. Consequently, these categories should currently be considered investigational in lung cancer, pending prospective validation.

Similar challenges exist for HER2 amplification assessment. HER2 amplification status is primarily determined using fluorescence in situ hybridization (FISH), with amplification defined as a HER2/CEP17 ratio ≥ 2.0 based on analysis of at least 20 non-overlapping tumor nuclei [59,74]. Some clinical researchers further define high-level HER2 amplification using more stringent thresholds, including a HER2/CEP17 ratio ≥ 5 or an absolute copy number ≥ 10 [64]. However, these thresholds remain largely extrapolated from breast and gastroesophageal malignancies and have not been validated in SqCLC.

Chromogenic and dual in situ hybridization (CISH, DISH) methods may be considered acceptable alternatives, with high degrees of concordance with FISH results [77,78]. One of the major technical challenges to diagnosis occurs in cases of chromosome 17 polysomy or segmental duplications, where the HER2:CEP17 ratio < 2.0 despite an elevated absolute HER2 copy number, due to the parallel increase in both HER2 and CEP17 signal, leading to underestimation of amplification when ratio-based criteria are used alone [74]. Given the high prevalence of chromosomal instability and complex copy number alterations characteristic of SqCLC, reporting both HER2/CEP17 ratios and absolute HER2 copy number may improve interpretation and facilitate more consistent cross-study comparisons.

## 5. Therapeutic Landscape of HER2-Targeted Therapies in NSCLC

### 5.1. Mutation-Directed Therapeutic Strategies

Trastuzumab Deruxtecan (T-DXd) was the first FDA-approved drug in the treatment of HER2-mutant NSCLC based on a prespecified interim analysis of the DESTINY-Lung02 study at the approved dose of 5.4 mg/kg (n = 102), with an ORR of 50% (95% CI, 39.9–60.1) at final analysis, median DOR of 12.6 months (95% CI, 6.4–NE), and median PFS of 10.0 months (95% CI, 7.7–15.2) in previously treated HER2-mutant NSCLC [19].

Recently, the emergence of selective HER2 tyrosine kinase inhibitors (TKIs) has significantly broadened the therapeutic options available to patients with HER2-mutant NSCLC. Previously, pan-HER inhibitors such as afatinib, dacomitinib, and neratinib were only modestly effective with response rates of 0% to 13% [19,34]. In addition, other agents such as pyrotinib and poziotinib showed moderate efficacy with response rates (19–53%) but have been associated with significant EGFR-related toxicities, particularly grade 3+ diarrhea (17–26%) and grade 3+ rash (47–49%) [19].

Newer-generation HER2-selective TKIs have demonstrated higher response rates in HER2-mutant NSCLC, with an ORR of 60–71% in patients with TKD mutations. The only available irreversible HER2 TKI currently available for oral administration is Zongertinib. In the BEAMION LUNG-1 trial (cohort 1, n = 75), the ORR was 71% (95% CI, 60–80) among patients with HER2 TKD mutations who had been previously treated, and these patients had a median duration of response and median progression-free survival of 14.1 months (95% CI, 6.9–NE) and 12.4 months (95% CI, 8.2–NE), respectively, with a median follow-up 11.3 months [33].

Sevabertinib is a reversible inhibitor targeting mutant HER2 and mutant EGFR. In the recently conducted SOHO-01 trial, patients with HER2 TKD mutations who received previous treatment (cohort D, n = 81) achieved an ORR of 64% (95% CI, 53–75), had a median duration of response of 9.2 months (95% CI, 6.3–13.5) and had a median progression-free survival of 8.3 months (95% CI, 6.9–12.3), with a median follow-up 13.8 months. Furthermore, efficacy was observed in patients previously treated with HER2-targeted therapies (cohort E, n = 55), with a response rate of approximately 38% (95% CI, 25–52), median DOR of 8.5 months (95% CI, 5.6–16.4), and median PFS of 5.5 months (95% CI, 4.3–8.3); median follow-up: 11.7 months [34]. In this trial, the response rate of patients with YVMA insertion mutation in HER2 exon 20 was substantially higher than those with other HER2 variants [34]. The optimal sequencing between these agents is currently undefined [68].

Both Zongertinib and Sevabertinib have obtained accelerated FDA approval for HER2 TKD mutations and are now considered standard-of-care second-line therapeutic options for non-squamous NSCLC patients with HER2 TKD mutations who have received prior systemic therapy alongside T-DXd [33,34]. The FDA very recently approved zongertinib as a first-line therapy option for patients with advanced or metastatic NSCLC with HER2 mutations, making it the first HER2-directed TKI. First-line data from BEAMION LUNG-1 cohort 2 showed an ORR of 76% (95% CI, 65–84) in 74 patients, with a median DOR of 15.2 months (95% CI, 9.8–NE) and median PFS of 14.4 months (95% CI, 11.1–NE); median follow-up for PFS: 15.2 months [33]. Sevabertinib also showed first-line activity in SOHO-01 cohort F (n = 73): ORR of 71% (95% CI, 59–81) with a median DOR of 11.0 months (95% CI, 8.1–NE); median PFS data were immature at the primary analysis (median follow-up: 9.9 months), although it has not been FDA approved as first line [34].

Although both Zongertinib and Sevabertinib have demonstrated clinical efficacy in HER2 TKD mutations, their activities in non-TKD mutations are only modest. In exploratory cohorts of non-TKD mutations, zongertinib showed an ORR of 30% (95% CI, 15–52), lower than responses observed in TKD-mutant populations. Data of Sevabertinib in non-TKD HER-2 mutant patients are very limited. In the SOHO-01 study, Sevabertinib showed an ORR of 14% (1/7) among pretreated HER2 therapy-naïve patients, 33% (1/3) among post-ADC patients, and 100% (2/2) among treatment-naïve patients, although sample sizes were very small. This is clinically important because FDA approvals for both agents are restricted to TKD-activating HER2 mutations [34].

This distinction may be particularly relevant in SqCL, where extracellular domain (ECD) mutations, including S310F/Y, appear relatively enriched compared with adenocarcinoma. Because HER2-selective TKIs target the intracellular kinase domain, their applicability to ECD-mutant tumors may be inherently limited. In contrast, antibody- drug conjugates (ADCs), including trastuzumab deruxtecan (T-DXd), utilize extracellular HER2 binding and intracellular payload delivery mechanisms that are less dependent on mutation location and therefore may provide broader activity across mutation classes. In DESTINY-Lung01 (n = 91), responses were observed in patients with mutations across all three exon locations including exon 20 TKD insertions, exon 19 TKD single-nucleotide variants, and exon 8 ECD mutations such as S310F/Y, ORR 55% (95% CI, 44–65), median DOR 9.3 months (95% CI, 5.7–14.7), median PFS 8.2 months (95% CI, 6.0–11.9), and median follow-up 13.1 months [19]. Consequently, T-DXd currently represents the most broadly applicable HER2-targeted strategy for HER2-mutant SqCLC, particularly for tumors harboring ECD mutations, where currently approved HER2-selective TKIs may have limited efficacy. However, there are currently no prospective clinical trial data for T-DXd specifically in HER2-mutant SqCLC. The pivotal trials that led to T-DXd’s FDA approval for HER2-mutant NSCLC, including DESTINY-Lung01, DESTINY-Lung02, and the ongoing DESTINY-Lung04, all exclusively enrolled patients with non-squamous histology. Therefore, therapeutic recommendations in HER-mutant SqCLC remain largely extrapolated from non-squamous datasets. Table 3 summarizes current HER2-directed therapeutic strategies according to HER2 alteration class, while highlighting the extent to which available evidence is derived from non-squamous cohorts and extrapolated to SqCLC.

Across all pivotal trials, squamous cell carcinoma patients are either explicitly excluded (Beamion LUNG-1, DESTINY-Lung03) or effectively absent (DESTINY-Lung01/02, where adenocarcinoma accounts for 98–100% of enrolled patients).

### 5.2. Amplification- and Expression-Driven Therapeutic Strategies

HER2 amplification and protein overexpression are two different therapeutic targets from activating HER2 mutations. Antibody-drug conjugates (ADCs) target cell-surface HER2 expression, and they are the most relevant treatments for this subset [19].

Unlike tyrosine kinase inhibitors, which depend on oncogenic activation of the HER2 kinase domain, ADCs use the presence of the HER2 protein expressed on the cell surface for targeted intracellular delivery of cytotoxic payload [23]. The antibody component binds to HER2 on the cell surface, resulting in receptor-mediated internalization and lysosomal degradation, releasing a potent cytotoxic agent within the tumor cell [19,23,80]. Amplification and strong overexpression may enhance ADC uptake due to the increased density of HER2 receptor [19]. Additionally, some ADCs, such as trastuzumab deruxtecan (T-DXd), have also been shown to demonstrate a bystander effect via utilization of a cleavable linker and a membrane-permeable topoisomerase I inhibitor payload, with a high drug-to-antibody ratio, thus permitting the diffusion of released cytotoxic agents into nearby tumor cells, irrespective of the level of HER2 expression [19,81,82].

Clinical evidence supporting the use of ADC is derived primarily in non-squamous NSCLC. In the DESTINY-Lung01 trial, T-DXd demonstrated a meaningful antitumor effect in patients with HER2-overexpressing NSCLC (IHC2+/3+) who had been previously treated, with an ORR of approximately 34% in the 5.4 mg/kg dose cohort [23]. Interestingly, in patients with IHC3+ tumors who received T-DXd, the ORR approached 50% or greater, suggesting a quantitative correlation between levels of protein overexpression and therapeutic benefit [23]. Antitumor activity was observed in patients with and without HER2 amplification, indicating that IHC-defined protein overexpression is the more reliable predictor of ADC response [23,83]. Beyond mutation-directed approvals, the tumor agnostic approval has important implications for SqCLC. These findings were the basis of the April 2024 FDA tumor agnostic approval of T-DXd at 5.4 mg/kg based on results from DESTINY-PanTumor02 for patients with unresectable or metastatic HER2-overexpressing (IHC3+) solid tumors who have progressed following prior treatment and who have no satisfactory alternative treatment options, including but not specifically for NSCLC, irrespective of histologic subtype [23,83]. Because the trial was conducted exclusively in patients with non-squamous histology, however, it did not provide any specific data for those with squamous cell carcinoma at this juncture [83]. Currently, there have been no published, prospective clinical trials specifically evaluating HER2-directed ADCs in patients with SqCLC with HER2 protein amplification or overexpression.

In terms of safety and tolerability, the most significant adverse effect associated with T-DXd is interstitial lung disease (ILD), which has been reported in approximately 5% of patients who received T-DXd 5.4 mg/kg. The rate of development of ILD has been shown to be dose dependent, with a rate of approximately 20% in patients receiving 6.4 mg/kg dose [23]. Other toxicities include neutropenia, nausea, a decline in left ventricular ejection fraction, significant fatigue, and alopecia [84].

Using naked monoclonal antibodies, such as trastuzumab, to target HER2 in patients with NSCLC has been largely unsuccessful. The combination of trastuzumab with chemotherapy in patients with HER2-positive NSCLC resulted in no improvement in survival. The apparent lack of benefit observed in these studies is likely a result of lower levels of HER2 expression as well as the high level of heterogeneity in HER2 expression in NSCLC. In addition, the cytotoxic effects of anti-HER2 antibodies are insufficient alone to block the signaling [23]. Ado-trastuzumab emtansine (T-DM1) showed modest efficacy as a HER2-targeting agent, resulting in an approximate ORR of 44%; however, the duration of response to T-DM1 is shorter. T-DM1 is currently considered a viable treatment alternative in this patient population according to NCCN guidelines [68].

### 5.3. Mechanisms of Resistance

Resistance to HER2 therapies can occur through HER2-dependent and -independent mechanisms [85,86,87]. HER2-dependent mechanisms involve alterations at the level of the HER2 receptor itself, which include HER2 copy number alterations, acquired secondary mutations (e.g., HER2 T798I gatekeeper, T862A, L755S), truncated HER2, and other mechanisms that might reduce target availability and limit the effectiveness of HER2-targeted therapies [85,86]. Loss of HER2 amplification and gene expression was also confirmed as a resistance mechanism in preclinical models [85].

HER2-independent mechanisms comprise activation through alternative pathways (e.g., MET amplification, PIK3CA mutations, epithelial-to-mesenchymal transition, and acquisition of cancer stem cell-like features) that can mediate resistance response [85,86,87]. DNA damage repair pathways alterations, as well as mutations affecting SWI/SNF chromatin-remodeling complex and other epigenetic regulators have also been implicated in promoting resistance to HER2 therapy [86]. EMT and acquisition of cancer stem cell-like features have been established as resistance mechanisms to HER2-targeted therapy in NSCLC [85]. PIK3CA mutations are well-established bypass pathway activators in targeted therapy resistance. MET amplification is another HER2-independent mechanism, which may restore downstream signaling pathways. Combined treatment with HER2 and MET inhibitors continues to produce benefit for patients with MET amplification who are resistant to other HER2 targeted agents [85].

Recently T-DXd-specific resistance mechanisms have been studied by Nilsson et al. and three distinct mechanisms of acquired T-DXd resistance were found, including payload resistance mediated by SLFN11 loss, copy number gains in the efflux pump ABCC1/MRP1, and secondary HER2 extracellular domain IV mutations [88].

Therapeutic approaches to overcome resistance to HER2 targeted therapy include sequential therapy with HER2-targeted agents, combination therapies that target bypass signaling pathways, and development of next-generation ADCs that demonstrate activity despite the presence of resistance-associated mutations. The observed activity of zongertinib and sevabertinib following progression on T-DXd suggests that there is no cross-resistance between ADCs and TKIs [34,89]. Conversely, T-DXd retains activity after prior HER2 TKI therapy. In the DESTINY-Lung01 trial, efficacy was observed across different subgroups, including those who had previously been treated with a HER2 tyrosine kinase inhibitor, although zongertinib and sevabertinib were not included. This bidirectional lack of cross-resistance supports the sequential use of ADCs and TKIs in either order. However, the optimal sequencing strategy may depend on HER2 alteration class. For tumors, harboring TKD mutations, particularly exon 20 insertions, sequential use of HER2-selective TKIs and ADCs represents a biologically rational approach given the demonstrated activity of both classes [19]. In contrast, for tumors enriched in ECD alterations, ADCs may be preferentially prioritized because currently approved HER2-selective TKIs demonstrate reduced and inconsistent activity outside the kinase domain; in the Beamion LUNG-1 exploratory cohort of non-TKD mutations, only 30% of patients achieved a confirmed objective response, and both zongertinib and sevabertinib carry FDA-approved indications restricted to TKD mutations [16,33]. Similarly, resistance mechanisms may vary by therapeutic class; secondary kinase domain mutations may preferentially impact TKI efficacy, whereas alterations affecting receptor internalization, lysosomal trafficking, or payload, including SLFN11 loss, ABCC1/MRP1 copy number gains, and secondary HER2 extracellular domain IV mutations at the trastuzumab binding site, may preferentially influence ADC activity [88]. Importantly, preclinical and clinical data suggest that tumors with acquired T-DXd resistance through these payload- or antibody-binding mechanisms maintain HER2 signaling and remain sensitive to HER2 TKIs [88]. Therefore, resistance mechanisms and HER2 alteration class should be considered jointly when selecting subsequent therapy.

## 6. HER2 Alterations and Immunotherapy in Squamous NSCLC

Platinum-doublet chemotherapy combined with immune checkpoint inhibitors (ICIs) remains the standard of care in advanced non-small cell lung cancer, including the squamous cell subtype unless contraindicated [68]. These recommendations apply irrespective of HER2 status, as there is no prospective evidence currently available to support modifying first-line chemo-immunotherapy based on HER2 alterations in squamous histology.

Overall response to ICIs in NSCLC varies based on HER2 alteration type. HER2-mutant tumors have moderate sensitivity to ICI monotherapy, with reported objective response rates of approximately 7–27% and median PFS of 4–7 months [19]. The German National Network Genomic Medicine study reported an ORR of 16% and median PFS of 4 months for ICI monotherapy in second or subsequent lines (n = 34) [90]. There may also be variation within mutation subtype; some studies suggest longer progression-free survival among tumors with non-exon 20 variants compared with exon 20 insertions [91]. Non-exon 20 insertion patients also had more concurrent mutations and higher TMB, which may contribute to enhanced immunotherapy sensitivity [91]. However, the LC-SCRUM-Asia study (n = 268) found no significant difference in clinical outcomes of chemo-ICI vs. chemo-alone by HER2 mutation subtype (median PFS 8.5 vs. 6.3 months; HR 0.66), suggesting this finding may not be consistent across all datasets [92].

Similarly, HER2 amplification appears to be associated with limited response to immunotherapy. In retrospective studies of HER2-amplified NSCLC treated with ICI monotherapy, objective response rates approached 0%, and median PFS approximately 2 months [93]. There was a lack of response even in tumors with high PD-L1 expression or elevated tumor mutational burden, suggesting that HER2 amplification may represent a subset with relative immune resistance [93]. While the majority of the available data derive from non-squamous populations, squamous patients included in these cohorts displayed a similar lack of differential benefit.

Importantly, interpretation of immunotherapy outcomes in HER2-altered SqCLC requires substantial caution, as most available evidence is derived from non-squamous or mixed histology cohorts in which squamous tumors are underrepresented or entirely excluded. Furthermore, biologic differences characteristic of SqCLC and distinct tumor microenvironments between squamous and non-squamous NSCLC may limit direct extrapolation from adenocarcinoma populations [94]. A meta-analysis of 12 studies (260 patients) reported a pooled ORR of 26% and median PFS of 5.36 months with ICIs in HER2-mutant NSCLC, with improved outcomes when combined with chemotherapy, but again without squamous-specific analyses [95]. Consequently, current observations regarding immunotherapy responsiveness in HER2-altered disease should be considered hypothesis generating rather than definitive for SqCLC.

## 7. Emerging Precision Medicine Strategies

Recent advances in antibody-drug conjugates, next-generation HER2 tyrosine kinase inhibitors, bispecific antibodies, and dual-target platforms are expanding the therapeutic landscape for HER2-altered NSCLC.

### 7.1. Next-Generation AntibodyDrug Conjugates

Beyond trastuzumab deruxtecan, other ADCs are emerging to expand therapy, overcome resistance, and enhance tumor penetration [19,68]. Trastuzumab rezetecan, a next-generation ADC, provides a cleavable tetrapeptide-based linker, and a novel topoisomerase I inhibitor payload (SHR169265) with a drug-to-antibody ratio of 6:1, that is designed to enhance cytotoxicity [52]. Trastuzumab rezetecan has an overall response rate of 73% and a median PFS of 11.5 months in patients with HER2-mutant NSCLC in the phase II HORIZON-LUNG trial, representing one of the highest response rates for a registered HER2-targeted study to date [52]. Responses were observed across multiple HER2 mutation subtypes and in patients previously treated with HER2 TKIs [52]. However, published data were derived predominantly from non-squamous populations, and SqCLC-specific enrollment characteristics and outcomes have not been separately reported. Therefore, the applicability of these results to HER2-altered SqCLC remains uncertain.

### 7.2. Bispecific Antibody Approaches

Another emerging treatment for HER2 is bispecific antibodies. The recently developed bispecific antibody, zanidatamab, binds to two different HER2 epitopes to promote HER2 receptor clustering and internalization, thus enhancing the effect on tumor cell death [96]. Although zanidatamab is currently approved for HER2-positive biliary tract cancer, this dual-epitope strategy may have potential applications in HER2-altered lung cancers [96].

In addition, dual targeting methods are being developed to overcome intratumoral heterogeneity. A novel bispecific ADC targeting HER2 and TROP2 (BIO-201) has shown significant preclinical antitumor activity [97]. Since approximately 82–90% of NSCLC tumors are TROP2+, including squamous histology, dual targeting of HER2 and TROP2 could broaden therapeutic coverage in tumors with heterogeneous HER2 expression [98,99]. Evidence for clinical activity of TROP2-directed ADCs such as datopotomab deruxtecan and sacituzumab govitecan in NSCLC already exists, further supporting the biological rationale for combination or dual-target therapy [98].

### 7.3. Combination Strategies

Combination therapies are also under investigation to enhance therapeutic durability and overcome resistance. Ongoing studies are evaluating ADC-immunotherapy combinations, including Trastuzumab deruxtecan with PD-1/PD-L1 inhibitors such as durvalumab or pembrolizumab. Trials such as DESTINY-Lung03, U106, and HUDSON are currently evaluating whether these combinations could result in greater antitumor immune responses and improved clinical outcomes [23,100].

Although these trials have predominantly enrolled patients with non-squamous histology and currently lack SqCLC-specific efficacy data, the biologic rationale supporting HER2-directed approaches remains relevant to squamous tumors.

#### Ongoing Clinical Trials

Several ongoing clinical trials are evaluating HER2-targeted therapies across multiple solid tumors types, including NSCLC. Due to the rarity of HER2 alterations in SqCLC, most studies feature ‘tumor agnostic’ designs where patients are enrolled based on molecular alterations rather than histologic subtype. Future clinical trials should therefore incorporate dedicated SqCLC cohorts or histologic stratification within HER2-directed studies to better define efficacy according to mutation class and histologic subtype. Ongoing trials continue to evaluate next-generation HER2 TKIs, ADCs, bispecific antibodies, and combination strategies designed to improve response durability and overcome resistance mechanisms. Table 4 summarizes selected ongoing clinical trials in HER2-directed therapies relevant to NSCLC.

## 8. Future Directions and Conclusions

Future progress in HER2-altered squamous NSCLC will depend on improved molecular characterization and dedicated clinical investigation. Large-scale genomic initiatives and collaborative datasets, including expansions of existing resources such as AACR Project GENIE, LC-SCRUM, and future TCGA-derived squamous focused datasets, will be critical to better define the prevalence, mutation spectrum, co-mutation landscape, and therapeutic vulnerabilities unique to HER2-altered SqCLC [53,101]. From a clinical perspective, broader adoption of reflex comprehensive genomic profiling at diagnosis of advanced SqCLC, including evaluation of ERBB2 exons 8,19 and 20, may improve identification of clinically relevant HER2 alterations that remain underrecognized in squamous histology. In parallel, translational studies examining the tumor microenvironment, immune contexture, and resistance mechanisms are essential for determining how HER2 signaling influences therapeutic response and for identifying biomarkers that refine patient selection [86,92].

A major barrier to progress in HER2-altered SqCLC remains the persistent underrepresentation of exclusion of squamous histology within HER2-directed clinical trials. Continued extrapolation from adenocarcinoma-dominant datasets is unlikely to adequately address the distinct biology of HER2-altered SqCLC. Therefore, future HER2-directed clinical development should move beyond pooled NSCLC enrollment strategies and incorporate histologic stratification or dedicated SqCLC cohorts as a predefined design element. Sponsors, cooperative groups, and regulatory agencies should prioritize prospective inclusion of SqCLC patients within ongoing and future HER2-directed programs, including DESTINY-Lung studies, BEAMION platforms, SOHO successor studies, and tumor-agnostic basket trials, to enable meaningful efficacy analyses according to histology and mutation subtype. Beyond conventional HER2 mutation and amplification testing, emerging biomarkers may further refine patient selection for next-generation HER2-directed therapies in SqCLC. Increasing attention has focused on HER2-low and HER2-ultralow expression states, particularly given the bystander effect associated with antibody-drug conjugates; however, the predictive significance of these categories remains undefined in GEA criteria for SqCLC [66,102]. Additional biomarkers under investigation include circulating tumor DNA (ctDNA) dynamics for treatment monitoring and minimal residual disease assessment [103,104]. In the sevabertinib trial, early ctDNA clearance was associated with prolonged progression-free survival (14.7 months) compared with persistent ctDNA detection (7.0 months), independent of initial radiographic response [34]. ctDNA-based MRD detection has also demonstrated strong prognostic value in resected NSCLC, with postoperative ctDNA positivity associated with significantly shorter disease-free survival (HR 3.45) and overall survival (HR 2.82) [105,106]. HER3 co-expression is under investigation as a potential mediator of resistance and therapeutic vulnerability; co-occurring gain-of-function mutations in HER2 and HER3 enhance HER2/HER3 activation and confer resistance of HER2-targeted therapies, which can be reversed by combined treatment with PI3K α inhibitors [107]. HER3 alterations co-occur with HER2 alterations at a significantly higher rate than expected (8.5% vs. 3.9% in HER2 wild-type, *p* < 0.001), and HER3-directed ADCs such as patritumab deruxtecan have shown intracranial and extracranial activity in NSCLC regardless of driver mutation status [108]. GPC3 and TROP2 are both highly expressed in lung squamous cell carcinoma, and dual-targeted approaches have shown potent preclinical antitumor activity [109]. Integration of these biomarkers into future clinical studies may facilitate more precise patient selection and improve therapeutic personalization in HER2-altered SqCLC [110].

Several important limitations should be acknowledged when interpreting the current evidence surrounding HER2-altered SqCLC. First, this review is narrative in nature and therefore reflects the limitations inherent to available published data. Second, most evidence informing HER2 biology, therapeutic efficiency, and biomarker development originates from non-squamous or mixed histology cohorts, with limited prospective data specifically evaluating SqCLC populations. Third, because HER2 alterations remain uncommon in SqCLC, many reported findings are derived from small retrospective analyses, exploratory subgroup evaluations, or tumor-agnostic studies, limiting definitive conclusions regarding treatment efficacy and prognostic significance in squamous histology. Consequently, many recommendations discussed herein remain extrapolative and require prospective validation in dedicated SqCLC cohorts.

In conclusion, HER2 alterations represent a rare but potentially actionable molecular subset of squamous NSCLC. While significant therapeutic advances have been achieved in HER2-mutant lung adenocarcinoma, the clinical relevance of HER2-targeted therapies in squamous histology remains incompletely defined. From a practical clinical perspective, comprehensive molecular testing should be strongly considered in patients with advanced SqCLC, including broad next-generation sequencing and HER2 expression assessment when clinically appropriate. For patients with HER2-mutant or HER2-overexpressing (IHC 3+) tumors, trastuzumab deruxtecan currently represents the most broadly applicable HER2-directed therapeutic strategy, particularly given its activity across multiple HER2 alteration classes and its tumor agnostic regulatory approval. In contrast, currently approved HER2-selective tyrosine kinase inhibitors primarily target kinase domain alterations and may have limited applicability in SqCLC due to the relative enrichment of extracellular domain mutations. Whenever feasible, enrollment in HER2-directed basket studies, tumor-agnostic trials or dedicated SqCLC cohorts should be prioritized to expand the evidence base for this rare molecular subset.

## Figures and Tables

**Table 1:** HER2 mutations in NSCLC, associated receptor domains, biological effects, and therapeutic implications.

Variant	Domain	Predominant Histology	Biologic Effect	Drug Sensitivity/Therapeutic Implications
Y772A775dupYVMA (A775G776insYVMA) [33,34]	Exon 20 in-frame insertion; TKD αC-helix–β4 loop	Lung adenocarcinoma; never smokers, women, and Asian populations.	Most common exon 20 insertion (48–62% of HER2 ex20ins in NSCLC). Enhances HER2 kinase activity via enhanced autophosphorylation and transphosphorylation of EGFR an HER3, activating the MAPK and PI3K-AKT pathways. Stabilizes the active kinase conformation and creates steric hindrance within ATP-binding pocket.	Resistant to pan HER TKIs (afatinib; dacomitinib). Clinical activity observed with HER2-selective TKIs: (Zongertinib, Sevabertinib), T-DXd, poziotinib, pyrotinib.
G776delinsVC [35]	Exon 20 deletion/insertion; TKD Gly776 region	Primarily reported in lung adenocarcinoma; uncommon variant	Constitutive TKD activation; reduces sensitivity to HER2 inhibitors such as lapatinib. Preserves drug-binding pocket geometry (no steric hindrance, unlike YVMA).	More sensitive to afatinib than YVMA. Clinical activity observed with Pyrotinib, T-DXd and HER2-selective TKIs.
P780Y781insGSP (G778P780dup) [33]	Exon 20 in-frame insertion; TKD αC-β4 loop	Predominantly adenocarcinoma; relatively uncommon exon 20 insertion subtype	Constitutive TKD activation; reduces sensitivity to lapatinib. Less conformational restriction than YVMA; preserves TKI affinity.	More sensitive to pan-HER TKIs than YVMA. Poziotinib ORR 71% in this subtype. Zongertinib ORR 75%. Clinical activity observed with Neratinin basket trials and T-DXd.
**L755S** [33,36]	Exon 19 missense (TKD)	More commonly reported in breast cancer; uncommon in NSCLC; usually described in adenocarcinoma	Constitutive activation; when acquired as secondary mutation, enhances HER2/MAPK signaling and promotes acquired resistance.	Resistant to lapatinib and neratinib (as secondary mutation). Preclinical activity reported with Osimertinib and Poziotinib active in preclinical models. Durable responses reported with Zongertinib; (20.5 mo) reported in L755P variant.
V777L [37]	Exon 20 missense; TKD near αC-helix	Primarily adenocarcinoma associated	Activates mutation; increases HER2/MAPK signaling. Oncogenicity may depend partly on cooperating mutations (e.g., PIK3CA).	Resistant to lapatinib. Sensitive to irreversible TKIs including neratinib. Clinical activity observed with and T-DXd.
D769H/D769Y [37]	Exon 20 missense; TKD	Rare; predominantly in adenocarcinoma and pan-tumor cohorts	Activates mutations; increased intrinsic kinase activity. Reduces sensitivity to lapatinib.	Resistant to lapatinib, gefitinib. Sensitive to neratinib. Clinical activity reported with Pyrotinib + T-DM1 combination, and T-DXd (DESTINY-PanTumor01 eligible).
V842I [23,37,38]	Exon 21 missense; TKD activation loop	Rare; predominantly in adenocarcinoma and pan-tumor cohorts	Activating mutation; promotes constitutive kinase activation and receptor pre-dimerization.	Sensitive to neratinib. Clinical activity preserved with T-DXd; (DESTINY-PanTumor01). Lapatinib sensitivity may be preserved.
S310F [34,39]	Extracellular domain (ECD) subdomain II	Relatively enriched in SqCLC compared with TKD mutations; associated with smoking, higher TMB, and frequent co-mutations (e.g., KRAS, STK11, KEAP1)	Most frequent ECD mutation (51% of ECD mutations in NSCLC). Promotes covalent homodimerization; activates the PI3K/AKT and MAPK pathways. Can form active heterodimers with EGFR. Reduces activity of trastuzumab and lapatinib.	Limited activity reported with HER2-TKIs (sevabertinib: 1/7 non-TKD patients responded in cohort D). ADC-based approaches preferred: T-DXd ORR 29.4% across tumor types (DESTINY-PanTumor01). Cetuximab may inhibit S310F-EGFR heterodimers.
G309E [35]	Extracellular domain (ECD) subdomain II	Rare ECD mutation, enriched in smoking-associated tumors including SqCLC	Activates mutation; induces receptor activation similar to S310F.	Limited TKI sensitivity. ADC-based approaches preferred.
Ile655Val [32,40]	Transmembrane domain (TMD)	Most extensively studied in breast cancer risk and trastuzumab toxicity research	Germline polymorphism rather than somatic driver mutation. Enhances dimerization and activation of HER2.	Val isoform-expressing tumors sensitive to trastuzumab. No established role for TKI therapy.
V659E/G660D [41]	Transmembrane domain (TMD)	Rare; reported across tumor types; limited NSCLC data	Activate TMD mutations; stabilize receptor dimerization and signaling. V659E is the most common TMD mutation.	Anti-HER2 antibodies and small-molecule kinase inhibitors block TMD/JMD mutant activity. Clinical responses to zongertinib and HER2 blockade have been reported.

Abbreviations: ADC, antibody–drug conjugate; AKT, protein kinase B; ATP, adenosine triphosphate; ECD, extracellular domain; EGFR, epidermal growth factor receptor; ex20ins, exon 20 insertion; HER, human epidermal growth factor receptor; JMD, juxtamembrane domain; MAPK, mitogen-activated protein kinase; NSCLC, non-small cell lung cancer; ORR, objective response rate; PI3K, phosphoinositide 3-kinase; SqCLC, squamous cell lung cancer; T-DM1, ado-trastuzumab emtansine; T-DXd, trastuzumab deruxtecan; TKD, tyrosine kinase domain; TKI, tyrosine kinase inhibitor; TMB, tumor mutational burden; TMD, transmembrane domain.

**Table 3:** Clinical evidence for HER2-directed therapies in HER2-altered NSCLC: efficacy, histologic eligibility, and evidence applicability to squamous cell carcinoma.

HER2 Alteration Class	Drug	Trial	N (Total)	Histology Allowed	N Squamous Cases	Line of Therapy	ORR	DoR	PFS	CNS Activity	Key Toxicity	FDA/NCCN Status	Squamous-Specific or Extrapolated?
HER2 mutation (predominantly exon 20 insertion)	Trastuzumab deruxtecan (T-DXd) 5.4 mg/kg q3w	DESTINY-Lung01 (Cohort 2) [19]	91	Adenocarcinoma predominant; squamous not reported	0 (NR)	2nd line+	55%	9.3 mo	8.2 mo	ORR 54.5% in stable BM subgroup (n = 33); mPFS 7.1 mo	ILD 26% (2 fatal); neutropenia 19% ≥ G3	FDA accelerated approval (all NSCLC histologies); NCCN preferred 2L+	Extrapolated; no squamous cases reported
HER2 mutation (predominantly exon 20 insertion)	Trastuzumab deruxtecan (T-DXd) 5.4 mg/kg q3w	DESTINY-Lung02 (corrected final analysis) [79]	102 (5.4 mg/kg arm)	Adenocarcinoma 98%; squamous absent	0 (effectively excluded by demographics)	2nd line+	49%	16.8 mo	10.0 mo (95% CI 7.7–15.2)	Intracranial responses observed; comparable to overall cohort; consistent with DL01	ILD/pneumonitis (grade ≥ 3 higher at 6.4 mg/kg); nausea	Basis for approved 5.4 mg/kg dose FDA approved; NCCN preferred	Extrapolated; squamous patients effectively absent
HER2 mutation (TKD)	Zongertinib (TKI)	Beamion LUNG-1 Cohort 1 (2L+) [33]	75 (120 mg)	Non-squamous only (SCC in exploratory Cohort 3)	0 in Cohort 1; included in Cohort 3	≥2L	71%	14.1 mo	12.4 mo	Intracranial ORR 47% (Cohort 4, active BM)	G ≥ 3 TRAE 17%; no drug-related ILD	FDA accelerated approval (non-squamous); NCCN preferred 2L+	Extrapolated; SCC in exploratory cohort only (Cohort 3: ORR 30% for non-TKD + SCC combined)
HER2 mutation (TKD)	Zongertinib (TKI)	Beamion LUNG-1 Cohort 2 (1L) [33]	74	Non-squamous only	0	1L	76%	15.2 mo	14.4 mo	Intracranial ORR 47% (Cohort 4)	G ≥ 3 TRAE 19%; 2 G2 ILD cases	NCCN 1L option	Extrapolated
HER2 mutation (TKD)	Sevabertinib 20 mg PO BID	SOHO-01	73	Not clearly restricted; predominantly non-squamous	NR	2nd line+	64–71%	8–9 mo	8–9 mo	Limited data	Rash, diarrhea	Not FDA approved; NCCN other option	Extrapolated; insufficient squamous data
HER2 mutation (TKD)	Sevabertinib (TKI)	SOHO-01 Cohort F (1L) [34]	73	As above	NR	1L	71–75%	11.0–12.2 mo	13.5 mo (updated)	Not reported	Diarrhea 88% (G3 6%); G ≥ 3 TRAE 23–25%	Not yet FDA approved for 1L	Extrapolated
HER2 mutation	Trastuzumab rezetecan (ADC)	HORIZON-Lung (Phase 2) [52]	94	NSCLC (China-only)	NR	≥2L	73%	>2/3 ongoing at cutoff	11.5 mo	Activity in BM subgroup	Neutropenia 40% G3–4; ILD 5%	Not FDA approved; investigational	Extrapolated

Abbreviations: TKD = tyrosine kinase domain; T-DXd = trastuzumab deruxtecan; ORR = objective response rate; DoR = duration of response; PFS = progression-free survival; BM = brain metastases; ILD = interstitial lung disease; IHC = immunohistochemistry; NR = not reached/reported; mo = months; NCCN = National Comprehensive Cancer Network.

**Table 4:** Selected ongoing clinical trials evaluating HER2-directed therapies relevant to HER2-altered NSCLC.

Trial (NCT)	Phase	Therapy	Target/Mechanism	HER2 Alteration Type	Population	Key Eligibility	Recruitment Status *	SqCLC Eligible?	Primary Endpoint
NCT06581432 Beamion PANTUMOR-1	II	Zongertinib (BI 1810631)	Oral, irreversible, HER2-selective TKI; spares WT EGFR	Amplification; activating mutation (excludes HER2-mutant NSCLC cohort)	Advanced solid tumors with HER2 alterations; ≥1 prior line; 13 cohorts	HER2 amplification or activating mutation in non-NSCLC tumors; ≥18 years	Recruiting	Potentially, if SqCLC carries HER2 amplification/overexpression rather than mutation, but HER2-mutant SqCLC is excluded	ORR (per cohort)
NCT06126276 Combo-MATCH	II	Neratinib ± Palbociclib	Pan-HER TKI ± CDK4/6 inhibitor	Amplification (IHC 3+; or IHC 2+ with FISH/NGS+)	Advanced HER2+ solid tumors except breast cancer; ≤5 prior lines; no RB1 loss	ECOG 0–2; no prior HER2 TKI or CDK4/6 inhibitor; excludes breast cancer	Recruiting	Unspecified histology not restricted beyond breast cancer exclusion; SqCLC likely eligible but not stated	PFS
NCT05315700 ORIC-114 Study	I/II	ORIC-114	Brain-penetrant EGFR/HER2 inhibitor	Exon 20 insertion (EGFR or HER2); HER2 amplification/overexpression; atypical EGFR mutations (NSCLC only)	Advanced solid tumors; multiple NSCLC cohorts; CNS metastases allowed	≥1 prior line; measurable disease; ECOG 0–2	Recruiting	Unspecified, not explicitly restricted by histology	ORR
NCT06706076 BH-30643 Study (SOLARA)	I/II	BH-30643	Novel oral non-covalent macrocyclic OMNI-EGFR inhibitor	Kinase domain mutations (exons 18–21): EGFR classical, atypical, exon 20 insertion; HER2 mutations	Locally advanced or metastatic NSCLC with EGFR/HER2 kinase domain mutations	ECOG 0–1; measurable disease; pathologically confirmed NSCLC; prior standard therapy	Recruiting	Unspecified: NSCLC broadly enrolled; no histologic exclusion noted; SqCLC with HER2 mutation eligible	DLT/MTD (Phase I); ORR (Phase II)
NCT04589845 TAPISTRY	II	Trastuzumab emtansine (T-DM1) and other targeted agents	HER2-targeted ADC	Mutation	Advanced solid tumors with specific genomic alterations including HER2 mutations; tumor-agnostic	ECOG 0–2; HER2 mutation confirmed; NSCLC excluded from entrectinib cohort; other cohorts vary	Recruiting	Unclear: tumor-agnostic design; SqCLC eligibility cohort-dependent	ORR
NCT06293898 BL-M07D1 Study	I	BL-M07D1	HER2-targeted ADC	Overexpression (IHC 1–3+); amplification; activating mutation	Advanced HER2-expressing solid tumors; Cohort 7 for lung cancer; ≥2 prior lines	ECOG 0–1; LVEF ≥ 50%; ≥2 prior lines	Recruiting	Cohort 7 is lung cancer broadly; no histologic restriction specified	DLT/MTD; ORR
NCT06253871 IAM1363 Study	I/Ib	IAM1363	Novel oral irreversible Type II HER2-selective TKI (>5000-fold HER2 vs. EGFR selectivity)	Mutation; overexpression/HER2-positive	Advanced HER2-altered malignancies including NSCLC; brain metastases allowed	ECOG 0–1; LVEF ≥ 50%; relapsed/refractory HER2-altered malignancy	Recruiting	Unspecified: NSCLC included broadly; no histologic restriction noted; SqCLC likely eligible if HER2-altered	DLT/MTD; ORR
NCT06616766 YH42946 Study	I/II	YH42946	Novel oral TKI targeting HER2 aberrations and EGFR exon 20 insertions	Exon 20 insertion (HER2 or EGFR); amplification; overexpression	Advanced solid tumors (escalation); NSCLC with HER2 exon 20 insertion (expansion cohort)	ECOG 0–1; measurable disease; dose expansion restricted to NSCLC	Recruiting	Unspecified: NSCLC expansion cohort does not restrict by histology; SqCLC with HER2 exon 20 insertion potentially eligible	DLT/MTD (Phase I); ORR (Phase II)

SqCLC = squamous cell lung carcinoma; WT = wild-type; ADC = antibodydrug conjugate; TKI = tyrosine kinase inhibitor; ORR = objective response rate; PFS = progression-free survival; DLT = dose-limiting toxicity; MTD = maximum tolerated dose; IHC = immunohistochemistry; FISH = fluorescence in situ hybridization; NGS = next-generation sequencing. * Recruitment status verified via ClinicalTrials.gov as of June 2026.

## Data Availability

No new data were created or analyzed in this study. Data sharing is not applicable to this article.
